# A field survey on the dietary use of traditional Chinese medicine in selected regions with the Cantonese, Hakka, and Teochew populations in Guangdong province, China

**DOI:** 10.1002/fsn3.4295

**Published:** 2024-07-24

**Authors:** Jie‐wen Peng, Shao‐wei Chen, Ping Wang, Rui Huang, Qing Li, Zi‐hui Chen

**Affiliations:** ^1^ Department of Health Risk Assessment Research Center Guangdong Provincial Institute of Public Health Guangdong Provincial Center for Disease Control and Prevention Guangzhou Guangdong China; ^2^ Guangdong Provincial Institute of Public Health Guangdong Provincial Center for Disease Control and Prevention Guangzhou Guangdong China

**Keywords:** Cantonese, field survey, Guangdong province, Hakka, Teochew, traditional Chinese medicine

## Abstract

The purpose of this study was to assess the dietary consumption patterns of traditional Chinese medicine (TCM) among the population in Guangdong province. Employing a cross‐sectional design, the survey was conducted from 2019 to 2021 to examine the inclusion of TCM in the diet of participants in Guangdong province. Information regarding consumption of TCM during the preceding 12 months was collected using a food frequency questionnaire. The participant cohort comprised a total of 3031 participants, consisting of 1081 Cantonese, 967 Hakka, and 983 Teochew individuals. The survey included 42 TCM, with consumption rates ranging from 2.6% to 47.3%. The top five TCM comprised Citri Reticulatae Pericarpium (47.3%), Lonicerae Japonicae Flos (47.0%), Codonopsis Radix (46.4%), Polygonati Odorati Rhizoma (43.1%), and Siraitiae Fructus (41.5%), along with Panacis Quinquefolii Radix (41.5%). These TCM possess recognized therapeutic properties within TCM for clearing heat, drying dampness, and detoxification. Within the top decile of 10 TCM, only Lonicerae Japonicae Flos was ubiquitous across all three sub‐populations. Nonetheless, 11 TCM from the top 20 and 17 TCM from the top 30 overlapped among the three sub‐populations. The study revealed substantial variability in the consumption rates of different TCM. Notably, those with traditional Chinese medicine effects of clearing heat, drying dampness, and detoxification exhibited higher consumption rates. Disparities in the consumption rates of these TCM were noted among the Cantonese, Hakka, and Teochew populations.

## Graphical abstract

A comprehensive field survey encompassing 3031 participants was conducted. Consumption rates of 42 TCM ranged from 2.6% to 47.3%. Distinct disparities were identified across the Cantonese, Hakka, and Teochew populations.

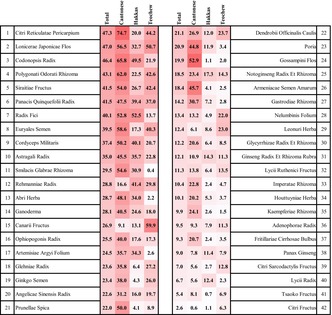

## 1 BACKGROUND

The concept of homology between traditional Chinese medicine (TCM) and food holds vital significance within Chinese traditions (Zhao et al., 2023). This homology emphasizes the capacity of botanical or zoological substances to provide nourishment and function as therapeutic agents in disease prevention and treatment (Hou & Jiang, 2013). In Chinese folklore, the distinction between herbal medicine and food was blurred, a practice also observable in Mediterranean, Europe, and global cuisines (Hu et al., 2022). The integration of TCM into dietary practices has been a historical and cultural norm in China, particularly evident in the use of edible and medicinal substances. TCM herbs are often used as ingredients in soups, teas, and even alcoholic beverages, believed to enhance their nutritional and medicinal properties. For instance, Ginseng and Goji berries are commonly added to teas for their perceived health benefits, while herbs like Astragalus and Codonopsis are popular soup ingredients (Commission CP, 2015, 2020).

According to the Food Safety Law of China, edible and medicinal substances are regulated through a designated list that is subject to dynamic management (The Central People's Government of the People's Republic of China, n.d.). Currently, this list includes 89 substances that are considered suitable for both dietary consumption and medicinal use, such as Citri Reticulatae Pericarpium (National Health Commission, n.d.). The list may be modified after a comprehensive evaluation conducted by the National Health Commission of the People's Republic of China and other relevant departments (National Health Commission, n.d.; National Health Commission of the People's Republic of China, 2021). For traditional foods that have unique local characteristics but are not covered by existing laws or the Chinese Pharmacopoeia, the establishment of provincial standards is encouraged among the different provinces of China (The Central People's Government of the People's Republic of China, n.d.).

Guangdong province in China is renowned for its rich culinary traditions, among which the practice of brewing “old fire soup” or “Lao Huo Liang Tang” stands out (Jia et al., 2010). This unique cooking method, deeply rooted in the Cantonese, involves slow‐cooking soups over low heat for extended periods, allowing the ingredients to release their full flavor and nutritional value (Zhang et al., 2016). In Guangdong, where the climate is often humid, soups brewed with TCM are particularly valued for their ability to nourish and harmonize the body. Ingredients like Poria and various tonic herbs are commonly used to strengthen the spleen, eliminate dampness, and enhance overall vitality (Wei et al., 2022). The belief is that regular consumption of these soups can help maintain health and prevent diseases, especially during the damp and rainy seasons.

Studies have extensively explored the diversity and utilization of therapeutic plant drugs in the traditional diets of the Guangdong's population (Ding et al., 2022; Liu et al., 2018; Lyu et al., 2021), yet conclusive data on consumption rates of commonly used TCM remain elusive. The aim of this study was to ascertain the consumption rate of mainly consumed TCM in the dietary habits of Guangdong's population through a large field study involving 3031 participants. Additionally, a comparative analysis was conducted to examine the consumption patterns of TCM among the Cantonese, Hakka, and Teochew populations within Guangdong province.

## 2 MATERIALS AND METHODS

### 2.1 Study design

A cross‐sectional study, conducted as a field survey from 2019 to 2021, aimed to explore the incorporation of TCM into culinary practices in Guangdong province, China. This study concentrated on the dietary consumption of TCM in three regions. Considering the significant population mobility and migration within Guangdong province, certain urban centers have become multicultural focal points, which could potentially influence local dietary traditions. For more accurate evaluation of TCM consumption, the Cantonese region was restricted to Guangzhou, Foshan, and Zhongshan cities; the Hakka region was limited to Meizhou, Heyuan, and Huizhou cities; and the Teochew region focused on Chaozhou, Shantou, and Jieyang cities (see Figure S1 for details). A probabilistic sampling methodology was utilized to ensure the inclusion of representative participants from the three designated areas. The participant selection process followed a procedure similar to our previous studies (Chen et al., 2019, 2021). In summary, three cities were selected from each of the Cantonese, Hakka, and Teochew populations, as mentioned previously. Within each city, personnel from the local Centers for Disease Control and Prevention (CDC) assisted in randomly selecting 1–3 counties. Subsequently, 2–4 communities or townships were randomly selected within each county. Finally, a random selection of 5–10 households was made within each community or township, and adult individuals were recruited for participation. The local CDC played a crucial role in the initial recruitment phase by providing us with a list of potential participants who met our age and residency criteria. The provincial team, which was responsible for conducting the household surveys, verified the basic information of the potential participants. This included confirming their age and the duration of their residence in the local area. To account for potential nonresponse or exclusion of participants during the verification process, we sampled approximately 110%–120% of the required number of survey subjects at each site.

### 2.2 Study population

The participants in this study were adults residing in the three selected regions of Guangdong province who had well‐established habits of consuming TCM in diet. The following inclusion criteria were applied in this study: (1) Participants must be aged 18 years or older and have a minimum residence of 6 months in the local area. (2) Participants were required to regularly incorporate TCM into their diet, such as using them as ingredients in soups or other food practices, as opposed to strictly medicinal usage. (3) Participants had to be in good health with no apparent medical conditions. The study targeted a minimum recruitment of 300 participants per city.

### 2.3 Inclusion of TCM

The questionnaire encompassed a thorough listing of 83 TCM, amalgamated from prior research on soup ingredients in Guangdong province and the official edible and medicinal substances list released by the National Health Commission of the People's Republic of China (National Health Commission, n.d.). According to a preliminary survey, several substances were excluded due to their extremely low consumption rate in Guangdong province. Nevertheless, owing to publication constraints and the study's focus, the analysis was confined to 42 TCM belonging to 29 families. These TCM can be ingested in various forms, such as soups, cooked dishes, teas, wines, preserves, or even consumed directly as a dietary component. The selection of these TCM was based on their elevated consumption rates among the Cantonese, Hakka, and Teochew populations within Guangdong province, comprising the top 30 TCM. Detailed information pertaining to the TCM, encompassing their Latin and Chinese nomenclature, edible parts, usage in dried or fresh form, and their medical applications (Table S1 displays the medical applications), was mainly sourced from the Chinese Pharmacopoeia (2020 edition) (Commission CP, 2020).

### 2.4 Food frequency questionnaire

A food frequency questionnaire (FFQ) was utilized to collect comprehensive data on the consumption of TCM over the preceding 12 months. Well‐trained investigators from local Centers for Disease Control and Prevention and/or community health service centers conducted personal interviews, diligently recording details of TCM consumption and their respective frequencies. To assist participants in recalling the types and frequencies of TCM consumed, we provided a color TCM spectrum during the interviews. This visual aid helped participants to identify and remember the specific plants they had consumed. Additionally, essential baseline information, such as age, gender, race, height, weight, education, and occupation, was documented.

### 2.5 Statistical analysis

Descriptive statistics, including the mean and standard deviation (SD), were used for continuous variables. Numbers and percentages were utilized to present discrete variables. Differences among groups in variables were compared by employing either a Student's *t*‐test or a chi‐square test, depending on the characteristics of the variables. Statistical analyses were performed utilizing R version 4.1.0, which was developed by the R Core Development Team. In this study, a *p*‐value less than .05 was considered statistically significant.

## 3 RESULTS

### 3.1 Characteristic of participants

The study included a total of 3031 participants, comprising 1081 Cantonese, 967 Hakkas, and 983 Teochew individuals. The average age of the participants was 46.4 ± 15.5 years, and males were accounting for 49.8% of the cohort. Across the three subgroups, no significant differences were found in variables including sex, race, height, weight, education, and occupation, except for average age. Detailed information is presented in Table 1.

**TABLE 1:** Characteristic of participants.

Variables	Cantonese (*n* = 1081)	Teochew (*n* = 983)	Hakkas (*n* = 967)	Overall (*n* = 3031)
Average age/years	50.7 ± 15.9	45.3 ± 15.0	42.7 ± 14.4	46.4 ± 15.5
Sex/*n*, %				
Male	542 (50.1)	482 (49.0)	485 (50.2)	1509 (49.8)
Female	539 (49.9)	501 (51.0)	482 (49.8)	1522 (50.2)
Race/*n*, %				
Han	1075 (99.4)	980 (99.7)	965 (99.8)	3020 (99.6)
Minority	6 (.6)	3 (.3)	2 (.2)	11 (.4)
Height/cm	162.6 ± 8.1	163.8 ± 8.1	163.8 ± 7.3	163.4 ± 7.9
Weight/kg	60.7 ± 10.4	61.3 ± 10.5	59.8 ± 10.6	60.6 ± 10.5
Education/*n*, %				
≤ 6 years	249 (23.0)	208 (21.2)	109 (11.3)	566 (18.7)
7–12 years	558 (51.6)	523 (53.2)	630 (65.1)	1711 (56.5)
≥ 13 years	274 (25.3)	252 (25.6)	228 (23.6)	754 (24.9)
Career/*n*, %				
Technical personnel	266 (24.6)	314 (31.9)	281 (29.1)	861 (28.4)
Business service	173 (16.0)	206 (21.0)	225 (23.3)	604 (19.9)
Housekeeper	154 (14.2)	152 (15.5)	219 (22.6)	525 (17.3)
Retiree	241 (22.3)	102 (10.4)	20 (2.1)	363 (12.0)
Agricultural and fishery technicians	79 (7.3)	35 (3.6)	35 (3.6)	149 (4.9)
Out of work	70 (6.5)	14 (1.4)	27 (2.8)	111 (3.7)
Student	15 (1.4)	24 (2.4)	23 (2.4)	62 (2.0)
Other	83 (7.7)	136 (13.8)	137 (14.2)	356 (11.7)

### 3.2 Consumption rate of TCM in three sub‐populations

**FIGURE 1:**
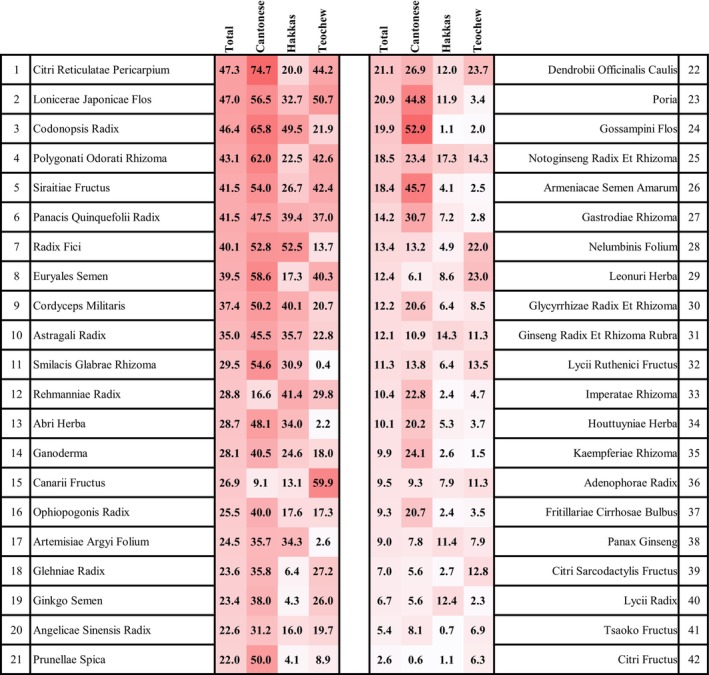
Consumption rates of 42 TCM in three sub‐populations.

The top five TCM in terms of overall consumption rates were as follows: Citri Reticulatae Pericarpium (47.3%), Lonicerae Japonicae Flos (47.0%), Codonopsis Radix (46.4%), Polygonati Odorati Rhizoma (43.1%), and Siraitiae Fructus (41.5%). Additionally, Panacis Quinquefolii Radix also exhibited a consumption rate of 41.5%. However, it is crucial to note that the consumption rates of TCM vary among the three sub‐populations, and the top five TCM differ in each sub‐population, as shown in Figure 1.

For Cantonese consumers, the five TCM with the highest consumption rates are as follows: Citri Reticulatae Pericarpium (74.7%), Codonopsis Radix (65.8%), Polygonati Odorati Rhizoma (62.0%), Euryales Semen (58.6%), and Lonicerae Japonicae Flos (56.5%).

For Hakka consumers, the five TCM with the highest consumption rates are as follows: Radix Fici (52.5%), Codonopsis Radix (49.5%), Rehmanniae Radix (41.4%), Cordyceps Militaris (40.1%), and Panacis Quinquefolii Radix (39.4%).

For Teochew consumers, the five TCM with the highest consumption rates are as follows: Canarii Fructus (59.9%), Lonicerae Japonicae Flos (50.7%), Citri Reticulatae Pericarpium (44.2%), Polygonati Odorati Rhizoma (42.6%), and Siraitiae Fructus (42.4%).

**FIGURE 2:**
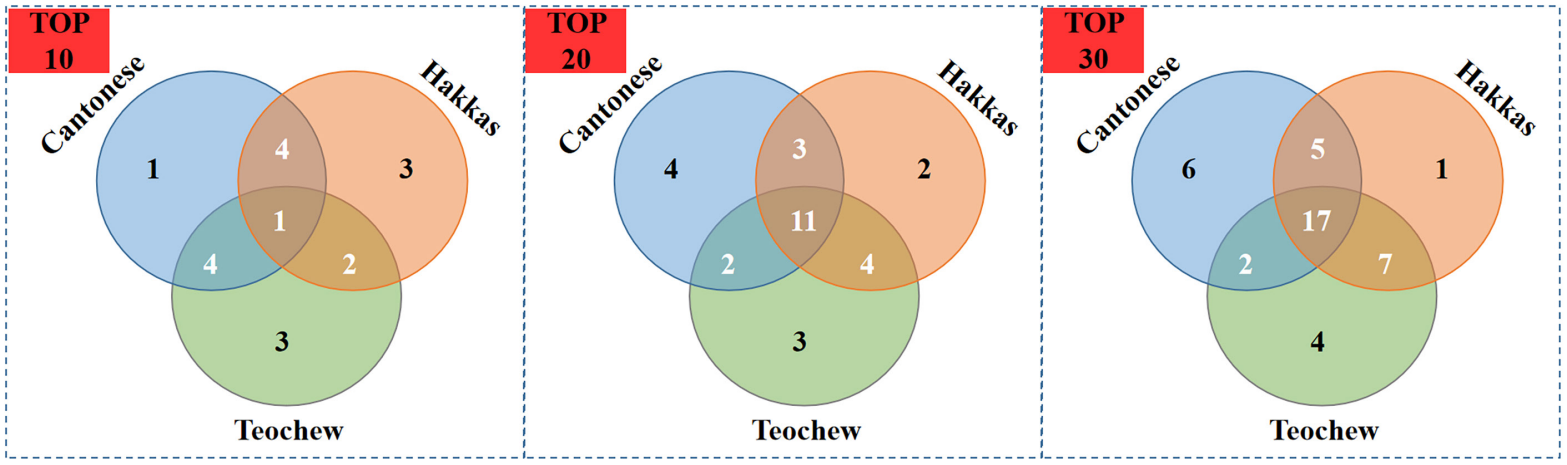
Comparison of top consumption rates in three sub‐populations.

When considering the top 10 consumption rates of TCM, it was observed that only one TCM, Lonicerae Japonicae Flos, consistently appeared within the top 10 lists across all three sub‐populations (Table 2). Nevertheless, there were 11 TCM from the top 20 and 17 TCM from the top 30 that overlapped among the three areas, as shown in Figure 2.

**TABLE 2:** Basic information of TCM used in three sub‐populations.

TCM name	Chinese name	Chinese character	Latin name of plant	Family	Part of plant	Dry/fresh	Specimen ID
Citri Reticulatae Pericarpium	Chen Pi	陈皮	*Citrus reticulata* Blanco	Rutaceae	Pericarp	Dry	GD‐01
Lonicerae Japonicae Flos	Jin Yin Hua	金银花	*Lonicera japonica* Thunb.	Caprifoliaceae	Flower	Dry	GD‐02
Codonopsis Radix	Dang shen	党参	*Codonopsis pilosula* (Franch.) Nannf.*, Codonopsis pilosula Nannf*. var. *modesta (Nannf.) L.D.Shen, Codonopsis tangshen Oliv*	Campanulaceae	Roots	Dry	GD‐03
Polygonati Odorati Rhizoma	Yu Zhu	玉竹	*Polygonatum odoratum (Mill.) Druce*	Asparagaceae	Roots and stems	Dry	GD‐04
Siraitiae Fructus	Luo Han Guo	罗汉果	*Siraitia grosvenorii (Swingle) C.Jeffrey ex A. M. Lu et Z. Y. Zhang*	Cucurbitaceae	Fruit	Dry	GD‐05
Panacis Quinquefolii Radix	Xi Yang shen	西洋参	*Panax quinquefolium* L.	Araliaceae	Roots	Dry	GD‐06
Radix Fici	Wu Zhi Mao Tao	五指毛桃	*Ficus hirta* Vahl	Moraceae	Roots	Dry	GD‐07
Euryales Semen	Qian Shi	芡实	*Euryale ferox* Salisb.	Nymphaeaceae	Seed	Dry	GD‐08
Cordyceps Militaris	Yong Chong Cao	蛹虫草	*Cordyceps militaris* (Linn, et Fr.) Link	Cordyceps	Stroma and fruiting body	Dry and fresh	GD‐09
Astragali Radix	Huang Qi	黄芪	*Astragalus membranaceus (Fisch.) Bge.var.mongholicus (Bge.) Hsiao, Astragalus membranaceus (Fisch.) Bge*.	Leguminosae	Roots	Dry	GD‐10
Smilacis Glabrae Rhizoma	Tu Fu Ling	土茯苓	*Smilax glabra* Roxb.	Smilacaceae	Roots and stems	Dry	GD‐11
Rehmanniae Radix	Sheng Di Huang	生地黄	*Rehmannia glutinosa* Libosch.	Plantaginaceae	Rhizome	Dry and fresh	GD‐12
Rehmanniae Radix Praeparata	Shu Di Huang	熟地黄	*Rehmannia glutinosa* Libosch.	Plantaginaceae	Rhizome	Dry	GD‐12
Abri Herba	Ji Gu Cao	鸡骨草	*Abrus cantoniensis* Hance	Leguminosae	Whole plant	Dry	GD‐13
Ganoderma	Ling Zhi	灵芝	*Ganoderma lucidum (Leyss.ex Fr.) Karst., Ganoderma sinense Zhao, Xu et Zhang*	Ganoderma	Fruiting body	Dry	GD‐14
Canarii Fructus	Qing Guo	青果	*Canarium album Raeusch*.	Burseraceae	Fruit	Dry	GD‐15
Ophiopogonis Radix	Mai Dong	麦冬	*Ophiopogon japonicus (L.f) Ker‐Gawl*.	Asparagaceae	Rhizome	Dry	GD‐16
Artemisiae Argyi Folium	Ai Cao	艾草	*Artemisia argyi* H. Lév. & Vaniot	Compositae	Leaf	Dry and fresh	GD‐17
Glehniae Radix	Bei Sha shen	北沙参	*Glehnia littoralis* F. Schmidt ex Miq.	Apiaceae	Roots	Dry	GD‐18
Ginkgo Semen	Bai Guo	白果	*Ginkgo biloba* L.	Ginkgoaceae	Seed	Dry and fresh	GD‐19
Angelicae Sinensis Radix	Dang Gui	当归	*Angelica sinensis (Oliv.) Diels*	Apiaceae	Roots	Dry	GD‐20
Prunellae Spica	Xia Ku Cao	夏枯草	*Prunella vulgaris* L.	Lamiaceae	Fruit cluster	Dry	GD‐21
Dendrobii Officinalis Caulis	Tie Pi Shi Hu	铁皮石斛	*Dendrobium officinale Kimura et Migo*	Orchidaceae	Stems	Dry and fresh	GD‐22
Poria	Fu Ling	茯苓	*Poria cocos (Schw.) Wolf*	Polyporaceae.	Sclerotium	Dry	GD‐23
Gossampini Flos	Mu Mian Hua	木棉花	*\Gossampinus malabarica (DC.) Merr*.	Malvaceae	Flower	Dry and fresh	GD‐24
Notoginseng Radix Et Rhizoma	San Qi	三七	*Panax notoginseng (Burk.) F. H. Chen*	Araliaceae	Roots and stems	Dry	GD‐25
Armeniacae Semen Amarum	Ku Xing Ren	苦杏仁	*Prunus armeniaca L. var. ansu Maxim., Prunus sibirica* L., *Prunus mandshurica (Maxim.) Koehne, Prunus armeniaca L*.	Rosaceae	Seed	Dry	GD‐26
Pruni Armeniacae Semen Dulce	Tian Xing Ren	甜杏仁	*Prunus sibirica* L.	Rosaceae	Seed	Dry	GD‐26
Gastrodiae Rhizoma	Tian Ma	天麻	*Gastrodia elata* Bl.	Orchidaceae	Rhizome	Dry	GD‐27
Nelumbinis Folium	He Ye	荷叶	*Nelumbo nucifera* Gaertn.	Nelumbonaceae	Leaf	Dry	GD‐28
Leonuri Herba	Yi Mu Cao	益母草	*Leonurus japonicus* Houtt.	Lamiaceae	Overground part	Dry and fresh	GD‐29
Glycyrrhizae Radix Et Rhizoma	Gan Cao	甘草	*Glycyrrhiza uralensis* Fisch. *Glycyrrhiza inflata* Bat., *Glycyrrhiza glabra* L.	Leguminosae	Roots and stems	Dry	GD‐30
Ginseng Radix Et Rhizoma Rubra	Hong shen	红参	*Panax ginseng* C.A. Mey.	Araliaceae	Roots and stems	Dry	GD‐31
Lycii Ruthenici Fructus	Hei Guo Gou Qi	黑果枸杞	*Lycium ruthenicum* Murray	Solanaceae	Fruit	Dry	GD‐32
Imperatae Rhizoma	Bai Mao Gen	白茅根	*Imperata cylindrica Beauv. var. major (Nees) C.E. Hubb*.	Poaceae	Roots and stems	Dry	GD‐33
Houttuyniae Herba	Yu Xing Cao	鱼腥草	*Houttuynia cordata* Thunb.	Saururaceae	Overground part	Dry and fresh	GD‐34
Kaempferiae Rhizoma	Shan Nai	山柰	*Kaempferia galanga* L.	Zingiberaceae	Roots and stems	Dry	GD‐35
Adenophorae Radix	Nan Sha shen	南沙参	*Adenophora stricta* Miq. *Adenophora tetraphylla* (Thunb.) Fisch.	Campanulaceae	Roots	Dry	GD‐36
Fritillariae Cirrhosae Bulbus	Chuan Bei Mu	川贝母	*Fritillaria cirrhosa* D. Don, *Fritillaria unibracteata* Hsiao et K.C. Hsia, *Fritillaria przewalskii* Maxim., *Fritillaria delavayi* Franch., *Fritillaria taipaiensis* P. Y. Li, *Fritillaria unibracteata* Hsiao et K. C. *Hsiavar. Wabuensis (S. Y. Tanget S. C. Yue) Z. D. Liu, S. Wang et S. C. Chen*	Liliaceae	Bulb	Dry	GD‐37
Panax Ginseng	Gao Li shen	高丽参	*Panax ginseng* C.A. Mey.	Araliaceae	Roots and stems	Dry	GD‐38
Citri Sarcodactylis Fructus	Fo Shou	佛手	*Citrus medica* L. var. sarcodactylis Swingle	Rutaceae	Fruit	Dry	GD‐39
Lycii Radix	Gou Qi Gen	枸杞根	*Lycium chinense* Mill.	Solanaceae	Roots	Dry	GD‐40
Tsaoko Fructus	Cao Guo	草果	*Amomum tsao‐ko* Crevost & Lemarié	Zingiberaceae	Fruit	Dry	GD‐41
Citri Fructus	Xiang Yuan	香橼	*Citrus medica* L.*, Citrus wilsonii* Tanaka	Rutaceae	Fruit	Dry	GD‐42

### 3.3 Monthly consumption frequency of TCM among consumers

Over 50% of the participants reported consuming 25 TCM less than once per month. The majority of the remaining TCM were mainly consumed either less than once per month or between 1.0 and 3.9 times per month. A small percentage of consumers reported consuming TCM four times per month or more times per month, as shown in Table 3.

**TABLE 3:** Monthly consumption frequency of TCM among consumers (*n*, %).

TCM name	*n*	<1.0 time/month	1.0–3.9 times/month	≥4.0 times/month
Citri Reticulatae Pericarpium	1434	430 (30.0)	670 (46.7)	334 (23.3)
Lonicerae Japonicae Flos	1425	661 (46.4)	581 (40.8)	183 (12.8)
Codonopsis Radix	1405	553 (39.4)	660 (47.0)	192 (13.7)
Polygonati Odorati Rhizoma	1307	500 (38.3)	634 (48.5)	173 (13.2)
Siraitiae Fructus	1259	615 (48.8)	476 (37.8)	168 (13.3)
Panacis Quinquefolii Radix	1258	683 (54.3)	446 (35.5)	129 (10.3)
Radix Fici	1214	730 (60.1)	431 (35.5)	53 (4.4)
Euryales Semen	1197	454 (37.9)	521 (43.5)	222 (18.5)
Cordyceps Militaris	1134	547 (48.2)	462 (40.7)	125 (11)
Astragali Radix	1061	393 (37.0)	485 (45.7)	183 (17.2)
Smilacis Glabrae Rhizoma	893	443 (49.6)	389 (43.6)	61 (6.8)
Rehmanniae Radix	872	543 (62.3)	284 (32.6)	45 (5.2)
Abri Herba	871	531 (61.0)	310 (35.6)	30 (3.4)
Ganoderma	853	581 (68.1)	221 (25.9)	51 (6.0)
Canarii Fructus	814	431 (52.9)	304 (37.3)	79 (9.7)
Ophiopogonis Radix	772	369 (47.8)	329 (42.6)	74 (9.6)
Artemisiae Argyi Folium	744	578 (77.7)	147 (19.8)	19 (2.6)
Glehniae Radix	716	271 (37.8)	358 (50.0)	87 (12.2)
Ginkgo Semen	709	480 (67.7)	200 (28.2)	29 (4.1)
Angelicae Sinensis Radix	686	400 (58.3)	242 (35.3)	44 (6.4)
Prunellae Spica	668	340 (50.9)	272 (40.7)	56 (8.4)
Dendrobii Officinalis Caulis	640	332 (51.9)	236 (36.9)	72 (11.3)
Poria	632	231 (36.6)	275 (43.5)	126 (19.9)
Gossampini Flos	603	368 (61.0)	194 (32.2)	41 (6.8)
Notoginseng Radix Et Rhizoma	561	278 (49.6)	170 (30.3)	113 (20.1)
Armeniacae Semen Amarum	559	212 (37.9)	245 (43.8)	102 (18.2)
Gastrodiae Rhizoma	430	286 (66.5)	120 (27.9)	24 (5.6)
Nelumbinis Folium	406	250 (61.6)	127 (31.3)	29 (7.1)
Leonuri Herba	375	253 (67.5)	116 (30.9)	6 (1.6)
Glycyrrhizae Radix Et Rhizoma	369	217 (58.8)	115 (31.2)	37 (10.0)
Ginseng Radix Et Rhizoma Rubra	367	254 (69.2)	82 (22.3)	31 (8.4)
Lycii Ruthenici Fructus	344	139 (40.4)	124 (36.0)	81 (23.5)
Imperatae Rhizoma	316	140 (44.3)	133 (42.1)	43 (13.6)
Houttuyniae Herba	305	175 (57.4)	110 (36.1)	20 (6.6)
Kaempferiae Rhizoma	301	163 (54.2)	101 (33.6)	37 (12.3)
Adenophorae Radix	288	125 (43.4)	136 (47.2)	27 (9.4)
Fritillariae Cirrhosae Bulbus	281	188 (66.9)	78 (27.8)	15 (5.3)
Panax Ginseng	272	190 (69.9)	73 (26.8)	9 (3.3)
Citri Sarcodactylis Fructus	212	116 (54.7)	65 (30.7)	31 (14.6)
Lycii Radix	204	109 (53.4)	87 (42.6)	8 (3.9)
Tsaoko Fructus	163	108 (66.3)	42 (25.8)	13 (8.0)
Citri Fructus	80	46 (57.5)	23 (28.8)	11 (13.8)

### 3.4 Consumed amount per serving of TCM among consumers

The average consumed amount per serving for the five TCM consumed in either dry or fresh form ranged from 10.2 to 82.6 g per serving. The average consumed amount per serving for the remaining TCM consumed in dry form ranged from 6.2 to 21.3 g, as shown in Table 4.

**TABLE 4:** Consumed amount per serving of TCM among consumers.

TCM name	*n*	Mean	SD	Median	Quartile
Citri Reticulatae Pericarpium	1434	6.4	5.0	5.0	3.0–10.0
Lonicerae Japonicae Flos	1425	10.5	8.9	10.0	5.0–12.0
Codonopsis Radix	1405	10.6	8.3	10.0	5.0–12.0
Polygonati Odorati Rhizoma	1307	10.4	8.3	10.0	5.0–12.0
Siraitiae Fructus	1259	10.4	8.8	10.0	5.0–14.0
Panacis Quinquefolii Radix	1258	6.9	5.9	5.0	3.0–10.0
Radix Fici	1214	18.0	14.5	15.0	10.0–20.0
Euryales Semen	1197	16.9	16.8	10.0	5.0–20.0
Cordyceps Militaris	1134	11.3	7.7	10.0	5.0–15.0
Astragali Radix	1061	9.4	7.1	10.0	5.0–10.0
Smilacis Glabrae Rhizoma	893	19.1	15.2	15.0	10.0–25.0
Rehmanniae Radix	872	10.2	5.8	10.0	5.0–15.0
Abri Herba	871	16.3	13.5	10.0	10.0–20.0
Ganoderma	853	10.6	9.2	9.0	5.0–15.0
Canarii Fructus	814	14.9	11.6	12.0	10.0–16.0
Ophiopogonis Radix	772	10.0	7.7	10.0	5.0–10.0
Artemisiae Argyi Folium	744	82.6	76.0	60.0	30.0–100.0
Glehniae Radix	716	10.5	9.5	10.0	5.0–10.0
Ginkgo Semen	709	18.3	15.1	14.0	8.0–25.0
Angelicae Sinensis Radix	686	8.1	6.2	7.0	5.0–10.0
Prunellae Spica	668	13.4	11.8	10.0	5.0–18.0
Dendrobii Officinalis Caulis	640	8.7	7.6	6.0	4.0–10.0
Poria	632	11.2	10.4	10.0	5.0–12.0
Gossampini Flos	603	12.6	10.8	10.0	5.0–15.0
Notoginseng Radix Et Rhizoma	561	7.8	6.8	5.0	3.0–10.0
Armeniacae Semen Amarum	559	7.1	5.0	5.0	3.0–10.0
Gastrodiae Rhizoma	430	9.4	6.5	8.0	5.0–11.8
Nelumbinis Folium	406	12.9	15.1	10.0	5.0–15.0
Leonuri Herba	375	29.4	22.5	20.0	10.0–50.0
Glycyrrhizae Radix Et Rhizoma	369	6.5	5.9	5.0	3.0–10.0
Ginseng Radix Et Rhizoma Rubra	367	7.3	5.1	5.0	3.0–10.0
Lycii Ruthenici Fructus	344	7.2	6.0	5.0	3.0–10.0
Imperatae Rhizoma	316	14.7	12.4	10.0	5.5–20.0
Houttuyniae Herba	305	13.3	13.2	10.0	5.0–15.0
Kaempferiae Rhizoma	301	7.7	8.6	5.0	3.0–10.0
Adenophorae Radix	288	9.0	4.8	10.0	5.0–10.0
Fritillariae Cirrhosae Bulbus	281	6.2	6.1	5.0	3.0–10.0
Panax Ginseng	272	7.1	6.1	5.0	3.0–10.0
Citri Sarcodactylis Fructus	212	11.1	10.9	10.0	5.0–12.3
Lycii Radix	204	21.3	13.8	20.0	10.0–30.0
Tsaoko Fructus	163	10.9	14.9	5.0	3.0–10.0
Citri Fructus	80	8.7	7.5	6.5	5.0–10.0

## 4 DISCUSSION

This study represents a pioneering effort in examining the dietary consumption of TCM from the Cantonese, Hakka, and Teochew populations of Guangdong province, with a substantial participant cohort of 3031 individuals. The findings of this study indicate that substances, such as Citri Reticulatae Pericarpium, Lonicerae Japonicae Flos, and Codonopsis Radix, exhibited a high prevalence of consumption, with rates surpassing 45% among residents. Furthermore, the consumption rates demonstrated significant variation across these cultural areas. Our findings provided valuable information for the development of a comprehensive database detailing edible and medicinal substances. This database is instrumental as a cornerstone for facilitating the conduct of safety risk assessments on foods characteristic of the region and aids in the formulation and ongoing refinement of provincial food safety standards.

The study, conducted among the population of Guangdong, demonstrated significant variations in the rates of TCM consumption. Of the 42 surveyed TCM, the consumption rates ranged from 2.6% to 47.3%. The three TCM with the highest consumption rates were Citri Reticulatae Pericarpium, Lonicerae Japonicae Flos, and Codonopsis Radix. Citri Reticulatae Pericarpium, originating from the tangerine peel (Citrus reticulata Blanco), is characterized by the presence of hesperidin (C_28_H_34_O_15_), nobiletin (C_21_H_22_O_8_), and tangeritin (C_20_H_20_O_7_) (Gao et al., 2023; Li et al., 2023). The red or green tangerine peel is subjected to a drying process and subsequently stored for several years before use. Over the course of storage, Citri Reticulatae Pericarpium develops a unique fragrance and acquires a slightly spicy and bitter taste profile. According to TCM theory, Citri Reticulatae Pericarpium is purported to possess effects, such as regulating qi, invigorating the spleen, drying dampness, and eliminating phlegm. These effects are predominantly ascribed to the presence of its characteristic component and unique fragrance (Hao et al., 2021; Wang et al., 2022). In Guangdong province, Citri Reticulatae Pericarpium is frequently incorporated in the local diet and is versatile in its consumption forms, including soups, cooked dishes, teas, wines, preserves, or even eaten directly.

Notably, disparities in the consumption rates of these TCM were identified across the Cantonese, Hakka, and Teochew populations. Our findings corroborate those of previous studies (Ding et al., 2022; Liu et al., 2018; Luo et al., 2019). With a consumption rate range of 0.6%–74.7%, participants from the Cantonese population demonstrated a higher propensity to incorporate the 42 TCM into their diet. Nonetheless, diverse consumption rates were observed for these TCM in this population. The widespread appreciation for Cantonese slow‐cooked soup and Cantonese herbal tea, which often incorporate various TCM, is particularly evident in the Cantonese population, particularly in Guangzhou city. The longstanding practice of preparing and consuming slow‐cooked soup with TCM has been instrumental in elevating the consumption rates of these TCM among the Cantonese population (Ke, 2023). These observations indicate that the consumption rates of TCM are generally higher in the Cantonese population than those in the Hakka and Teochew populations.

The accessibility of TCM is a significant factor influencing consumption rates. The surveyed cities of Cantonese population, situated in the economically developed Pearl River Delta region of Guangdong province, have facilitated greater access to majority of TCM for the participants. This accessibility is likely a consequence of the availability of markets, herbal shops, including cultivation of such TCM within the region. Furthermore, differences in TCM principles among the populations in the three sub‐populations also contribute to the variations in consumption rates. Despite the fact that all three sub‐populations are from the East Asian monsoon region, notable differences persist in terms of climate and environment (Liang et al., 2020). The Teochew population, particularly from Shaotou city, boasts an extensive coastline, rendering its residents particularly vulnerable to the marine monsoon's influence (Li et al., 2017). On the other hand, the Hakka population is predominantly situated around mountainous areas, exposing them to cold valley winds. In contrast, the Cantonese population, being at lower latitudes, endures an extended summer period characterized by higher heat and humidity (Liu et al., 2013). These variations could significantly influence the incidence of specific health issues and the perceived efficacy of specific TCM in each region, thus affecting consumption rates among the population. The identification of these top TCM and the understanding of regional consumption patterns provide empirical references for selecting dosage standards of TCM in different regions. This is crucial for ensuring the safe and effective use of TCM in dietary practices and adds significant value to this research work. Future studies could delve deeper into the medicinal effects of these substances, their potential interactions, and the mechanisms underlying their popularity in specific regions.

The study's methodological rigor is enhanced by recognizing its inherent limitations. First, while the study focused on the Cantonese, Hakka, and Teochew areas, known for their distinct food cultures, it should be noted that participants from different sub‐populations may not strictly adhere to their local diet patterns due to the inevitable exchange and assimilation of food cultures. This could potentially introduce variability in dietary habits among participants. Second, the study did not thoroughly investigate and analyze the consumption of additional TCM in the local areas. The study employed a predefined list of TCM, which may have overlooked the consumption of other TCM that are more frequently consumed by the participants. Third, the study utilized a medium‐ to long‐term FFQ to assess dietary intake. Nevertheless, an analysis of factors, such as seasonal influences, TCM origins, and participant TCM constitutions, was not conducted in relation to consumption patterns. It is beyond the scope of this study to provide selection recommendations for the most suitable TCM for each season across the three sub‐populations. Finally, given that the study was premised on household surveys, this approach may result in an elevated recurrence rate of the same TCM consumption within households, thereby potentially biasing the overall data. These limitations should be taken into consideration when interpreting the results of the study, and further research may be warranted to explore these aspects in greater detail.

## 5 CONCLUSION

This study presents an analysis of the dietary consumption patterns of 42 TCM, surveying 3031 individuals from the Cantonese, Hakka, and Teochew populations in Guangdong province. Notably, Citri Reticulatae Pericarpium, Lonicerae Japonicae Flos, and Codonopsis Radix were prevalent, with a consumption rate of 45% among the sampled population, and are recognized in TCM for their therapeutic properties, including clearing heat, drying dampness, and detoxification. Variability was observed in the consumption rates of these TCM across diverse sub‐populations. These significant findings provide valuable insights into execution of safety risk assessments pertaining to the culinary traditions specific to each sub‐population, with implications for the development and enhancement of regulatory guidelines at the provincial level concerning food safety.

## AUTHOR CONTRIBUTIONS

**Jie‐wen Peng:** Conceptualization (equal); resources (lead); writing – review and editing (equal). **Shao‐wei Chen:** Conceptualization (lead); data curation (lead); investigation (equal); software (lead); writing – original draft (lead). **Ping Wang:** Data curation (equal); investigation (equal); methodology (equal); writing – review and editing (equal). **Rui Huang:** Data curation (equal); investigation (equal); methodology (equal); software (equal); writing – review and editing (equal). **Qing Li:** Investigation (equal); writing – review and editing (equal). **Zi‐hui Chen:** Conceptualization (lead); investigation (equal); project administration (equal); writing – review and editing (equal).

## FUNDING INFORMATION

This study received support from the Research Project of the Traditional Chinese Medicine Bureau of Guangdong Province (Grant No. 20212028), the Guangdong Provincial Key Research and Development Program (Grant No. 2019B020230001), and the Guangdong Mandatory Medical Research Fund Project (Grant No. C2019050).

## CONFLICT OF INTEREST STATEMENT

The authors declare that they have no competing interests.

## ETHICS STATEMENT

This study was performed in accordance with the Declaration of Helsinki.

## INFORMED CONSENT

Before being included in the study, all participants provided explicit and written consent after being fully informed of the study's objectives and procedures.

## Supporting information


Figure S1.

Table S1.

## Data Availability

The datasets used and/or analyzed during the current study available from the corresponding author on reasonable request.
