# Supplementary figures and images for: A field survey on the dietary use of traditional Chinese medicine in selected regions with the Cantonese, Hakka, and Teochew populations in Guangdong province, China
Source: Food Sci Nutr. 2024 Jul 24;12(10):7438–48. doi: 10.1002/fsn3.4295 (PMC11521733; doi:10.1002/fsn3.4295)

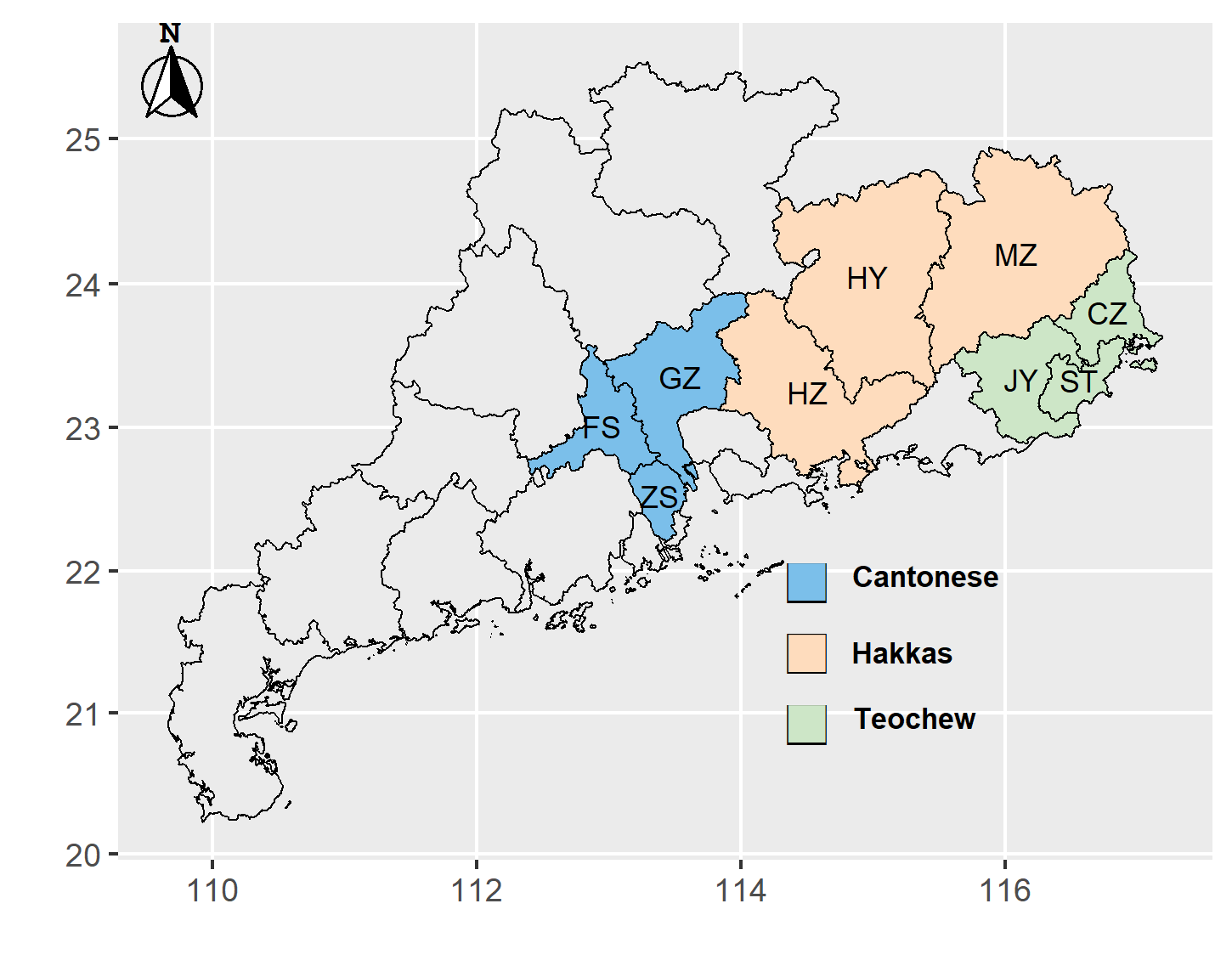

Supplement: Supplementary file 1 — Figure S1. [file FSN3-12-7438-s002.tiff]
