# Supplementary material for: A field survey on the dietary use of traditional Chinese medicine in selected regions with the Cantonese, Hakka, and Teochew populations in Guangdong province, China
Source: Food Sci Nutr. 2024 Jul 24;12(10):7438–48. doi: 10.1002/fsn3.4295 (PMC11521733; doi:10.1002/fsn3.4295)
Supplement: Supplementary file 2 — Table S1. [file FSN3-12-7438-s001.docx]

Table S1 Medical use of included TCM

Table S1 Medical use of included TCM

| **TCM name** | **Medical use** |
| --- | --- |
| Citri Reticulatae Pericarpium | Regulating qi and strengthening spleen, drying dampness and eliminating phlegm |
| Lonicerae Japonicae Flos | Clearing heat and detoxifying, evacuating wind and heat |
| Codonopsis Radix | Invigorating spleen and lung, nourishing blood and promoting fluid |
| Polygonati Odorati Rhizoma | Nourishing Yin moistening dryness, producing fluid to quench thirst |
| Siraitiae Fructus | Clearing heat and moistening lung, helping pharynx to open sound, smoothing bowel |
| Panacis Quinquefolii Radix | Tonifying qi and nourishing Yin, clearing heat and promoting fluid |
| Radix Fici | Supplementing qi and invigorating spleen, expelling phlegm and dampness, relaxing meridian and activating collaterals |
| Euryales Semen | Tonifying kidney and reinforcing essence, tonifying spleen and stopping diarrhea, dehumidifying and stopping belt |
| Cordyceps Militaris | Tonifying lung and kidney |
| Astragali Radix | Tonifying Qi rising Yang, strengthening the surface to stop perspiration, reducing swelling, promoting fluid and blood, stagnation Tongbi, supporting poison and discharging pus, restraining sores and producing muscle |
| Smilacis Glabrae Rhizoma | Detoxification, dehumidification, clearing joints |
| Rehmanniae Radix | Fresh Rehmannia clearing heat and promoting fluid, cooling blood and stopping bleeding Sheng Rehmannia clearing heat and cooling blood, nourishing Yin and promoting fluid. |
| Rehmanniae Radix Praeparata | Supplementing blood and nourishing Yin, supplementing essence and filling marrow |
| Abri Herba | Dampness-withdrawing yellow, clearing heat and detoxifying, soothing liver and relieving pain |
| Ganoderma | Tonifying qi and calming the mind, relieving cough and relieving asthma |
| Canarii Fructus | Clearing heat and detoxifying, benefiting pharynx and promoting body fluid |
| Ophiopogonis Radix | Nourishing Yin Sheng Jin, moistening lung clear heart |
| Artemisiae Argyi Folium | Warm channels to stop bleeding, dispel cold and pain; Remove dampness and relieve itching externally |
| Glehniae Radix | Nourishing Yin and clearing lung, benefiting stomach and promoting fluid |
| Ginkgo Semen | Reclining lung for asthma, stop belt shrinkage of urine |
| Angelicae Sinensis Radix | Tonifying blood and promoting blood circulation, regulating menstruation and relieving pain, moistening bowel |
| Prunellae Spica | Clear the liver and expel fire, clear the eyes, disperse the knot and detumescence |
| Dendrobii Officinalis Caulis | Benefiting stomach and promoting fluid, nourishing Yin and clearing heat |
| Poria | Moistening moisture, invigorating spleen and calming heart |
| Gossampini Flos | Clearing heat and dampness, detoxifying |
| Notoginseng Radix Et Rhizoma | Dissipate stasis to stop bleeding, reduce swelling and pain |
| Armeniacae Semen Amarum | Lowering qi to relieve cough and asthma, moistening bowel |
| Pruni Armeniacae Semen Dulce | Moisten lungs to relieve cough, moisten intestines |
| Gastrodiae Rhizoma | Calming wind and stopping spasm, calming liver-yang, expelling wind and clearing collaterals |
| Nelumbinis Folium | Clear summer-heat dampness, rise Qingyang, cool blood to stop bleeding |
| Leonuri Herba | Activating blood and regulating menstruation, diuresis and detumescence, clearing heat and detoxification |
| Glycyrrhizae Radix Et Rhizoma | Tonifying spleen and supplementing qi, clearing heat and detoxifying, expelling phlegm and relieving cough, relieving acute pain and harmonizing various medicines |
| Ginseng Radix Et Rhizoma Rubra | Big tonifying qi, compound pulse solid, qi and blood |
| Lycii Ruthenici Fructus | Clearing heart heat, strengthening kidney, moistening liver and brightening eyes, strengthening stomach and nourishing brain, anti-aging and menses |
| Imperatae Rhizoma | Cooling blood to stop bleeding, clearing heat diuresis |
| Houttuyniae Herba | Clearing heat and detoxification, eliminating carbuncle and discharging pus, diuretic and Tonglin |
| Kaempferiae Rhizoma | Temperature, digestion, pain relief |
| Adenophorae Radix | Nourishing Yin and clearing lung, benefiting stomach and promoting fluid, eliminating phlegm and benefiting Qi |
| Fritillariae Cirrhosae Bulbus | Clearing heat and moistening lung, resolving phlegm and relieving cough, clearing knot and eliminating carbuncle |
| Panax Ginseng | Big tonifying qi, compound pulse solid, qi and blood |
| Citri Sarcodactylis Fructus | Soothing the liver and regulating the qi, and stomach pain, dry dampness and phlegm |
| Lycii Radix | Remove dampness fire, withdraw vacuity heat |
| Tsaoko Fructus | Dry dampness temperature, stop malaria and eliminate phlegm |
| Citri Fructus | Soothing the liver and regulating the qi, widening the middle, eliminating phlegm |
